# Tinbergen’s four questions: Two proximate, two evolutionary

**DOI:** 10.1093/emph/eoy035

**Published:** 2018-12-06

**Authors:** Randolph M Nesse

**Affiliations:** Center for Evolution and Medicine, Arizona State University, PO Box 871701, Tempe AZ 85287–1701, USA

## DEFINITION AND BACKGROUND

In 1951, Ernst Mayr suggested that biology includes two enterprises that ask different questions.^1^ ‘Functional biologists’ ask ‘proximate questions’ about structures and ‘how’ mechanisms work. ‘Evolutionary biologists’ ask ‘ultimate questions’ about ‘why’ organisms are the way they are. The distinction between proximate and evolutionary explanations is a core principle of evolutionary medicine,^2^ but Mayr’s terminology has caused confusion.^3^ He called the study of proximate mechanisms ‘functional biology’, but studying the adaptive functions of traits is central to the evolutionary, not proximate explanations. Associations aroused by the word ‘ultimate’ led Mayr to later recommend using ‘evolutionary explanations’ instead.^4^ Also, ‘why questions’ can incorrectly imply teleology.

In 1963, the ethologist Niko Tinbergen expanded Mayr’s distinction into what are now known as ‘Tinbergen’s Four Questions’.^5^ He called them ‘causation, ontogeny, evolution and survival value’; now they are often referred to as ‘mechanism, ontogeny, phylogeny and adaptive significance’. The first two are proximate questions, the last two are evolutionary questions creating a 2 × 2 Table.^6^

## EXAMPLES IN CLINICAL MEDICINE AND PUBLIC HEALTH

Descriptions of the biochemical pathway that synthesizes bilirubin and how it develops in the individual provide both parts of a proximate explanation; describing the costs, benefits and the evolutionary history of that pathway provides both parts of an evolutionary explanation.

Fever is expressed in response to relevant cues, so the proximate explanation must describe the mechanisms that detect them and regulate fever, and those that adjust body temperature; their development is the other half of a full proximate explanation. The two parts of an evolutionary explanation are provided by describing how the capacity for fever gives a selective advantage, and the evolutionary history of the responsible mechanisms.

Seeking answers to all four of Tinbergen’s questions expands explanations beyond mechanisms to also describe a trait’s development, evolutionary history and adaptive significance. These answers can help to explain characteristics of a trait that make it vulnerable to malfunction.

**Table:** 

**Tinbergen’s Four Questions**		**Two objects of explanation**	
		**Sequence** (Diachronic)	**Single form** (Synchronic)
**Two kinds of explanation**	**Proximate**	**Ontogeny**	**Mechanism**
		How does the trait develop in individuals?	What is the structure of the trait?
	
	**Evolutionary**	**Phylogeny** What is the trait’s evolutionary history?	**Adaptive significance** How have trait variations influenced fitness?

**Conflict of interest** **:** None declared.
